# Lifestyle interventions for people with a severe mental illness living in supported housing: A systematic review and meta-analysis

**DOI:** 10.3389/fpsyt.2022.966029

**Published:** 2022-10-28

**Authors:** Lisanne E. M. Koomen, Marte Z. van der Horst, Jeroen Deenik, Wiepke Cahn

**Affiliations:** ^1^Department of Psychiatry, UMC Utrecht, Utrecht, Netherlands; ^2^Lister, Utrecht, Netherlands; ^3^GGnet, Warnsveld, Netherlands; ^4^GGz Centraal, Amersfoort, Netherlands; ^5^School for Mental Health and Neuroscience, Maastricht University, Maastricht, Netherlands; ^6^Altrecht, Utrecht, Netherlands

**Keywords:** lifestyle medicine, severe mental illness, physical activity, healthy eating, implementation, systematic review, meta-analysis

## Abstract

Although supported housing facilities (SHF) appear to be an ideal setting for supporting people with severe mental illness (SMI) to obtain a healthier lifestyle, little is known about the effects of lifestyle interventions in SHF and the factors contributing to successful implementation. We performed a systematic review and meta-analysis to assess the effect of lifestyle interventions on mental and physical health in people with SMI in SHF, and reviewed which intervention factors contribute to successful implementation. A meta-analysis using a random effects model was undertaken. Discussions were reviewed to identify factors that foster successful implementation. Of 7401 identified studies, 9 RCTs (*n* = 1260) were included for the systematic review and 8 (*n* = 1187) for the meta-analysis. Improvements in weight (*n* = 3), BMI (*n* = 1), 6-Min Walk Test (*n* = 1) and metabolic criteria (*n* = 2) were seen. In the meta-analysis we only found a small effect for a decrease in waist circumference. Reviewing factors involved with the implementation showed that the most successfully implemented interventions were multidisciplinary and integrated into standard care. In conclusion, we found limited evidence for the effectiveness of lifestyle interventions on physical health for those living in SHF. To reliably examine the effects on mental and physical health, more studies with high involvement of staff and participants are needed.

## Introduction

People with severe mental illness (SMI) often suffer from long lasting psychiatric symptoms and experience difficulties in rehabilitation. Despite current treatment options, two-thirds of patients experience long-term psychiatric symptoms that cause difficulties in day-to-day life (1–3) resulting in a lower quality of life (1). In addition, people with SMI have a 10–25 years lower life expectancy compared to the general population (4–8). The decreased life expectancy is largely due to a higher prevalence of cardiometabolic diseases (5, 7), which is mainly caused by the use of psychotropic drugs and an unhealthy lifestyle (i.e., smoking, dietary risks, and sedentary lifestyle) (4, 6, 9, 10). A meta-analysis (11) showed that in people with SMI, 50–65% were obese, 18–23% had hyperglycemia, 36% hypertriglyceridemia, 39% hypertension, and 39% low high-density lipoproteins (HDL) cholesterol. The prevalence of metabolic syndrome (MetS) was 33% and there was an increased risk of 58% on MetS compared to the general population.

Lifestyle interventions, such as promoting physical activity habits, healthy eating, and smoking cessation, can improve this impaired mental and physical health in people with SMI (12–18). Lifestyle interventions can reduce psychotic and mood symptoms, stimulate social functioning, increase quality of life, and there is early evidence that they can lower the need for psychotropic drugs (14, 15, 18, 19). Moreover, increased physical activity is associated with a decrease in all-cause mortality (20) and can reduce cardiometabolic risk factors (21).

However, in day-to-day practice, it is difficult for people with SMI to adopt and maintain a healthy lifestyle. Studies show that adherence to lifestyle interventions is low and high numbers of dropouts are reported (22–24). People with SMI experience several barriers, such as anxiety and negative symptoms, a lack of social support, knowledge, and money (25). Studies conducted in inpatient (26) and outpatient (27) settings show that there are also barriers at the staff and the organizational level that hamper successful implementation of lifestyle interventions. These barriers are a lack of time, understanding of the intervention, knowledge, and financial resources. The type of psychiatric and rehabilitation care people with SMI receive may have specific barriers and opportunities for lifestyle interventions. A significant number of people with SMI are living in supported housing facilities (SHF) and receive support in their rehabilitation and daily life. Despite the intensive and broad care that is provided, so far little attention has been paid to achieving and maintaining a healthy lifestyle. Nevertheless, SHF could be an ideal setting to support people with SMI in adopting a healthier lifestyle as people are living in their own environment and have less acute problems compared to people in inpatient care, but are guided in a more intensive way compared to an outpatient setting. Additionally, the mental health professionals in SHF are in close contact and work in a semi-intensive way with the people living in SHF and can therefore be of great value in overcoming the barriers (28).

Despite the evidence from meta-analyses on the effect of lifestyle interventions for people with SMI, no meta-analytic evidence exists for this effect in people with SMI living in SHF. This evidence would be valuable as the setting of SHF may present specific opportunities and barriers to lifestyle interventions for people with SMI and this evidence is needed to design and implement the most suitable lifestyle interventions and improve mental and physical health in this particular group and setting.

This study aimed to systematically evaluate lifestyle interventions for people with SMI in SHF to investigate which lifestyle interventions are effective in improving mental and physical health. Furthermore, we reviewed the discussions and the author’s recommendations to study which intervention factors contribute most to successful implementation and positive outcome of lifestyle interventions in this specific setting.

## Methods

We followed the PRISMA (Preferred Reporting Items for Systematic Review and Meta-Analyses) guidelines to ensure the quality of this systematic review and meta-analysis (29).

### Search strategy

The search was performed by two independent researchers (LK and MH). The databases Pubmed, Cochrane Database, PsychINFO, and Embase were systematically searched for randomized controlled trials from inception until July 2021. Combinations of the following (MeSH) terms were used: “exercise,” “lifestyle intervention,” “healthy lifestyle,” “sport*,” “physical activity,” “exercise therapy,” “diet,” “weight loss,” “stop smoking,” “quit smoking,” “smoking cessation,” “schizophrenia,” “psychosis,” “bipolar disorder,” “SMI,” “serious mental illness,” “severe mental illness.” SHF was not included in the search, but was a selection criterion in the full-text screening of the articles, so that no relevant articles were missed. The search results were limited to studies published in English or Dutch. There was no limit to date of publication. In Embase, conference papers from 2018, 2019 and 2020 were included in the search. Reference lists of relevant articles were manually examined. All manuscripts were imported into EndNote X7 software and duplicates were removed. Abstracts and titles were screened and relevant articles were retrieved for full text review.

### Inclusion criteria

Studies were included if the following criteria were met: (a) the paper reported a randomized controlled trial with a lifestyle intervention (any intervention that aims to improve lifestyle habits as physical activity, dietary habits, and sleeping pattern and/or smoking); (b) participants were diagnosed with SMI (defined as a psychiatric disorder classified by the DSM-5 that causes serious limitations in psychosocial functioning for a duration of ≥2 years) and at least 50% were living in SHF, to try to ensure that the lifestyle intervention was specifically targeted and designed for this particular group of patients, and (c) the paper was published in a peer-reviewed journal or conference book. Any comparator (e.g., treatment as usual, active comparator) and all outcomes on mental and physical health will be included.

Full text articles were reviewed by two independent researchers (LK and MH). In case of disagreement of inclusion, a third researcher was consulted to reach consensus.

### Data extraction

We extracted the following information from each study: authors, publication year, number of participants included in the intervention and control group, type of intervention, diagnoses of participants, living situation of participants, participant characteristics, and results. The majority of studies included participants who lived independently as well as in SHF. In these cases, the authors were emailed and requested for the data of the participants that lived in SHF, so that this specific data could be used for the analyses. If this data was not available, the number of participants living in SHF was used in the meta-analysis.

### Quality assessment

Two independent researchers (LK and MH) assessed the quality of the studies using the Cochrane Risk of Bias Tool (30). The risk of bias was assessed as “low,” “some,” or “high” (31). If study protocols were available, these were also screened for quality assessment.

### Strategy for meta-analyses

Meta-analysis was performed if at least three studies reported on the outcome measure. Comprehensive Meta-Analysis software was used. The random-effects model was used to analyze the data to account for the heterogeneity in study populations. For each individual study, Hedges’ g was calculated for each outcome measure in the meta-analysis. We calculated Hedges’ g in every treatment arm using the mean difference in change scores (end of treatment minus baseline) and standard deviations (SD) or pre- and post-means (± SD). We calculated all effect sizes to check for errors. Effect sizes with *p* < 0.05 were considered statistically significant. Hedges’ g’s of 0.20 were considered a small effect, 0.50 a medium effect, and over 0.80 a large effect (32). In addition, we performed sensitivity analyses on follow-up period, type of intervention (sports, psychoeducation), and sample size to examine sources of heterogeneity.

### Factors related to successful implementation

We reviewed the discussions and the author’s recommendations of each included paper to study which intervention factors contribute most to successful implementation and positive outcome of lifestyle interventions.

## Results

### Search results

**FIGURE 1:**
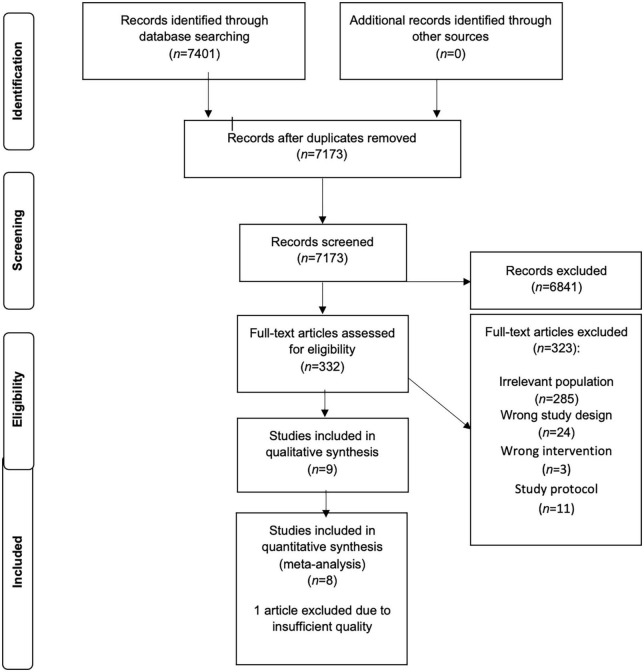
PRISMA flow chart.

A flow diagram of the literature search is displayed in Figure 1. 7401 articles were screened on title and abstract and 332 studies were retrieved for full text review. 9 articles fulfilled the inclusion criteria and were used in the analyses.

### Study quality

The results of the quality assessment are listed in Table 1. All studies scored ‘high concerns’ on deviations from the intended interventions and effect of assignment to intervention, since the instructors and participants were not blinded from the assigned intervention. We therefore chose to exclude this criterion from the quality assessment. In three studies (33–35) there were ‘high concerns’ of bias, as bias may have occurred due to missing outcome data or deviations from the intended intervention due to the study context. The study of Gyllensten and Forsberg (34) failed to implement the intervention, mainly due to staffing issues, and only 5% of the participants participated in the intervention. As a result, no reliable estimate can be made of the effectiveness of the intervention and it was decided to only include this study in the review on factors associated with successful implementation.

**TABLE 1:** Overview of the outcomes of the Cochrane Risk of Bias Tool (31).

Study	Randomization process	Deviations from the intended interventions (*effect of adhering to intervention*)	Missing outcome data	Measurement of the outcome	Selection of the reported result	Overall
Cabassa et al. (36)	Low	Low	Low	Low	Low	Low
Daumit et al. (40)	Low	Low	Low	Low	Low	Low
Forsberg et al. (28)	Some concerns	Low	Some concerns	Low	Low	Some concerns
Forsberg et al. (33)	Low	Low	High	Some concerns	Low	High
Gyllensten et al. (34)	Some concerns	High	High	Low	Low	High
Looijmans et al. (35)	Low	Low	High	Low	Low	High
Marzolini et al. (39)	Low	Low	Low	Low	Low	Low
Rotatori et al. (38)	Low	Low	Some concerns	Low	Low	Some concerns
Verhaeghe et al. (37)	Some concerns	Low	Some concerns	Low	Low	Some concerns

### Study and participant characteristics

Study and participant characteristics are depicted in Table 2. Eight studies (28, 33, 35–40) with a total number of 1187 participants examined the effect of lifestyle interventions on mental and/or physical health in people living in SHF. Forsberg et al. reported the effect of their lifestyle intervention in the same population in two different papers, one focusing on the effect on mental health (33) and one on physical health (28). Five studies (28, 33, 36–38), with a total number of 583 participants, only included participants living in SHF. The following types of interventions were implemented: psychoeducation on healthy lifestyle (*n* = 6) (28, 33, 36–38, 40), group sport sessions (*n* = 5) (28, 33, 37, 39, 40), meetings with an individual health coach or nurse (*n* = 1) (40), group sessions with behavioral techniques (*n* = 1) (36), and changing the environment into a more health and less obesogenic environment (*n* = 1) (35). The control conditions consisted of: care as usual (*n* = 4) (35–37, 39), in which in the study of Cabassa et al. control participants were also offered health promotion groups, waiting list condition (*n* = 1) (38), psychoeducation on nutrition, physical activity, and health (unrelated to weight) (*n* = 1) (40), or an aesthetic study circle (*n* = 2) (28, 33). The intensity of the interventions varied, with frequencies of group classes or sports sessions ranging from one (37), to two (28, 33, 38, 39), to three times a week (36, 40).

**TABLE 2:** Overview of study and participant characteristics.

Study	Study sample, *n*	Participants living in SHF, *n* (%)	Male, *n* (%)	Age (years), M (SD)	Diagnoses	BMI (kg/m^2^), M (SD)	Waist circum ference in (cm), M (SD)	Weight (kg), M (SD)	Intervention	Control	Outcome parameters	Main results	Follow-up period
Cabassa et al. (36)	314	314 (100)	181 (57)	48.7 (11.6)	Depression (*n* = 236, 75.2%), schizophrenia or schizoaffective disorder (*n* = 178, 56.7%), anxiety disorder (*n* = 158, 50.3%), bipolar disorder (*n* = 146, 46.5%), alcohol or drug use disorder (*n* = 121, 38.5%)	33.7 (7.2)	–	98.5 (24.3)	A total of 22 sessions of 60 min focusing on behavioral techniques to improve dietary habits and physical activity (*n* = 157) **Attendance:** The median of sessions attended was 18 of 22, with 59% of participants (*N* = 93) attending 50% of sessions, and 36% (*N* = 57) attending all 22 sessions.	Usual care, which also included health promotion groups (*n* = 157) **Attendance:** intervention and control participants did not differ in the use of usual care services	≥5% weight loss, cardiorespiratory fitness increase of ≥50 meters at the 6MWT, cardiovascular risk reduction defined as ≥5% weight loss or improvement of cardiorespiratory fitness	A larger proportion of the intervention group had weight loss at 12 and 18 months (not significant between groups). Both groups had weight loss from 6 to 18 months. No significant differences were found for mean weight loss and mean increases in 6MWT.	18 months
Daumit et al. (40)	291	159 (54.6)	145 (50)	45.3 (11.3)	Schizophrenia (*n* = 85, 29.2%) Schizoaffective disorder (*n* = 84, 28.9%) Bipolar disorder (*n* = 64, 22.0%) Major depression (*n* = 35, 12.0%) Other (*n* = 23, 7.9%)	36.3 (7.3)		102.7 (21.1)	Tailored group and individual weight-management sessions and group exercise sessions of moderate intensity (50 min per session, 3 times per week) (*n* = 144) **Attendance:** The median number of total attended sessions was 46 out of 62 in the first 6 months and 31 out of 164 in months 7 through 18.	Standard Nutrition and physical-activity information at baseline. Health classes quarterly, with content unrelated to weight. (*n* = 147)	Blood pressure BMI chemical levels Fasting blood glucose Waist circumference Weight	At 18 months, the net weight change was 3.2 kg (95% *CI* −5.1 to −1.2) in the intervention group in comparison to the control group.	18 months
Forsberg et al. (28)	41	41 (100)	25 (61)	41.3 (NS)	Schizophrenia (*n* = 23, 56.1%) Bipolar disease (*n* = 3, 7.3%) Other psychotic disease (*n* = 7, 17.1%) Other psychiatric diseases (*n* = 8, 19.5%)	30.3 (10.4)		Men 104.1 (19.2), women 77.1 (22.3)	Study circles on healthy food including cooking and sport classes, twice weekly for 2 h (*n* = 24) **Attendance:** 46.4% of the sessions	Aesthetic study circles, once a week for 2 h (*n* = 17) **Attendance:** 63.1% of the sessions	Blood pressure BMI Daily steps Exertion test HbA1C HDL Heart score Metabolic criteria Pulse rate Smoking habits Triglycerides Waist circumference Weight	A significant decrease in the number of metabolic criteria in the intervention group at 12 months in comparison to the control group (intervention: 3.00 vs. 2.24, *p* = 0.000. control: 2.00 vs. 2.08, *p* = 003).	12 months
Forsberg et al. (33)	41	41 (100)	25 (61)	41.3 (NS)	Schizophrenia (*n* = 23, 56.1%) Bipolar disease (*n* = 3, 7.3%) Other psychotic disease (*n* = 7, 17.1%) Other psychiatric diseases (*n* = 8, 19.5%)	30.3 (10.4)		Men 104.1 (19.2), women 77.1 (22.3)	Study circles on healthy food including cooking and sport classes, twice weekly for 2 h (*n* = 24) **Attendance:** 46.4% of the sessions	Aesthetic study circles, one a week for 2 h (*n* = 17) **Attendance:** 63.1% of the sessions	GAF MANSA SCL-90-R SF-36 SOC13	At 12 months increased SOC (mean change 8.4 in intervention vs. 0.6 in control, *p* = 0.05). Increase of 6.4 in GAF in intervention group (*p* = 0.041), but no between group difference. No effect on health-related QoL, subjective QoL, global level of functioning or change in symptoms.	12 months
Looijmans et al. (35)	736	434 (59)	465 (63)	48.3 (12.6)	Psychotic disorder (*n* = 534, 72.6%) Mood disorder (*n* = 76, 10.3%) Personality disorder (*n* = 238, 32.3%)	28.0 (6.3)	Men 104.4 (16.1), women 103.0 (17.0)		Creating a healthy environment for participants (*n* = 365) **Attendance:** not applicable due to the nature of the intervention	Care as usual (*n* = 371)	BMI HbA1C HDL LDL Metabolic Z-score Total cholesterol Triglycerides Waist circumference	At 3 months significant decrease of 1.51cm of waist circumference and metabolic syndrome z-score decreased by 0.22 (95% *CI* −0.38 to −0.06) in the intervention group compared to the control group. At 12 months decrease of 1.28cm of waist circumference, but no longer significant. No effect on metabolic z-score at 12 months. No significant differences in BMI.	12 months
Marzolini et al. (39)	13	11 (84.6)	8 (62)	44.6 (2.6)	Schizophrenia (*n* = NS) Schizoaffective disorder (*n* = NS)	28.3 (1.2)	102 (4.0)	82.1 (4.7)	Twice weekly exercise at a local recreation center (without specifications on duration or type of exercise) (*n* = 7) **Attendance:** mean 72%, all participants attended at least 50% of exercise classes	Care as usual (*n* = 6) **Attendance:** not applicable	6MWT Anthropometric measurements Blood pressure BMI MHI One repetition maximum test Waist circumference Weight	A non-significant increase of 27.7m on the 6MWT. Improvement in strength with the 1RM test for the exercise group (28.3 ± 8.8%, *p* = 0.01) but not for the control group (12.5 ± 8.5%, *p* = 0.2). There were no between group differences. Significant improvement in total MHI score for the exercise group (*p* = 0.03) with no significant improvement for the control group (*p* = 0.57). There were no between group differences.	12 weeks
Rotatori et al. (38)	14	14 (100)	6 (43)	35.9 (8.1)	Schizophrenia (*n* = NS) Alcohol use disorder (*n* = 2, 14.3%)				Twice weekly behavior therapy focusing on healthy lifestyle (*n* = 7) **Attendance:** not mentioned	Waiting list (*n* = 7)	Weight	Significant decrease in weight in the intervention group compared to the control group (mean change -3.3kg in intervention vs. + 2.54 in control, *p* ≤ 0.05).	7 months
Verhaeghe et al. (37)	284	284 (100)	174 (61)	46.4 (12.2)	Schizophrenia (*n* = 105, 71.3%) Mood disorder (*n* = 68, 51.6%) Substance misuse (*n* = 44, 32.4%) Personality disorder (*n* = 40, 28.2%) Other (*n* = 20, 16.5%)				Weekly psycho-educational and behavioral group sessions, supervised exercise, and individual support (*n* = 201) **Attendance:** 51.2% attended at least 8 of 10 sessions	Care as usual (*n* = 83)	BMI BSI Daily steps Dietary diary Fat mass IPAQ SF-36 Waist circumference Weight	Significant differences between the intervention and control group: At ten weeks in body weight (−0.35 vs. 0.22 kg, *p* = 0.04), BMI (−0.12 vs. 0.08 kg/m^2^, *p* = 0.04), WC (−0.29 vs. 0.55 cm, *p* < 0.01) and fat mass (−0.99 vs. −0.12%, *p* < 0.01) and mean steps per day (1256 ± 1933 steps/day vs. −426 ± 2754 steps/day, ≤ 0.001). At end point the decrease in the primary outcomes in the intervention group disappeared, with the exception of “fat mass” (33.76 vs. 34.17%). End point weight (88.28 vs. 87.95 kg), BMI (30.33 vs. 30.22 kg/m^2^) and WC (106.32 vs. 106.16 cm) were slightly above the baseline values. No effect on other outcomes.	9 months

BMI, Body Mass Index; BSI, Brief Symptom Inventory; IPAQ, International Physical Activity Questionnaire; GAF, Global Assessment of Functioning; M, Mean; MANSA, Manchester Short Assessment of Quality of Life; Metabolic Z-score, a standardized score for the cluster of five cardiometabolic risk factors; MHI, Mental Health Inventory; n, Number; NS, not stated; QoL, Quality of Life; ROM, Routine Outcome Monitoring; SCL-90-R, Symptom Checklist; SD, standard deviation; SF-36: Short Form Health Survey; WC, waist circumference; 1RM, One Repetition Maximum test; 6MWT, 6-Min Walk Test.

### Effect of lifestyle interventions on mental health and quality of life

#### Literature review

The effect of lifestyle interventions on mental health and quality of life was examined by three studies (28, 37, 39). Forsberg et al. (33) found an increase of 6.4 on the global assessment of functioning (GAF) score in the intervention group and Marzolini et al. (39) found a significant improvement on the mental health inventory for the intervention group, but both studies did not find between group differences. No significant effect on the other outcome parameters regarding mental health and social functioning was found.

#### Meta-analysis

Various non-comparable outcome measures were used for measuring psychiatric symptoms and social functioning and therefore this data could not be used in the meta-analysis. Two studies (33, 37) examined the effect of lifestyle interventions on quality of life and found no significant a significant effect.

### Physical health outcomes of lifestyle interventions

#### Literature review

Five studies (28, 35, 37, 39, 40) with a total of 516 participants examined the effect of lifestyle intervention on Body Mass Index (BMI). One study (37) found a significant decrease in BMI. The effect of lifestyle interventions on weight loss was studied in six studies (28, 36–40), of which three studies (37, 38, 40) found a significant decrease. No significant change in waist circumference was observed in any of the five studies (28, 35, 37, 39, 40). None of the studies found a significant change in blood pressure, total cholesterol, triglycerides, HDL cholesterol, LDL cholesterol, or HbA1C. Two (28, 35) of the three (28, 35, 36) studies that examined metabolic criteria did, however, find a significant decrease in metabolic criteria (glucose level, HDL level, triglyceride level, blood pressure and waist circumference). Lastly, two studies (36, 39) with a total number of 325 participants examined the effect on physical fitness using the 6 min Walking Test (6MWT) and in the study of Marzolini et al. (39) a superior effect over controls was found.

#### Meta-analysis

**FIGURE 2:**
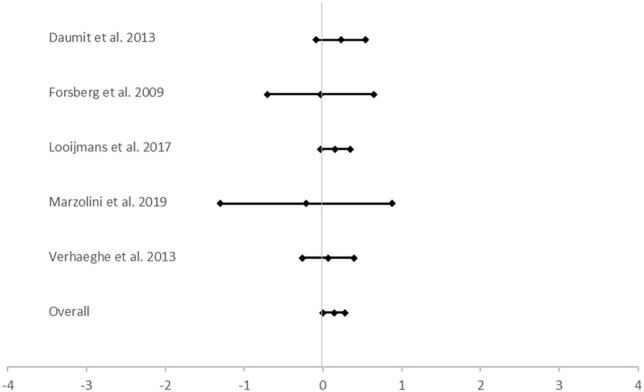
Meta analysis on the effect of a lifestyle intervention on waist circumference. Hedge’s g<0 favors control and Hedge’s g>0 favors intervention.

The meta-analysis showed a small significant reduction in waist circumference (Figure 2). On the outcomes BMI, weight, systolic blood pressure, triglycerides, HDL cholesterol and metabolic criteria no significant effect was found (Table 3).

**TABLE 3:** Overview of results of the meta-analyses.

Outcome measure	Studies (*n*)	Participants (*n*)	Hedges’ *g*	95% *CI*	*P*-value	I^2^ (%)
BMI	5	516	0.149	−0.113 to 0.412	0.264	36.561
Weight	6	816	0.185	−0.135 to 0.505	0.258	65.416
Waist circumference	5	811	0.142	0.001–0.284	**0.048**	0
Blood pressure						
*systolic*	4	572	0.017	−0.256 to 0.291	0.902	40.461
Triglycerides	3	459	0.120	−0.064 to 0.304	0.202	0
HDL	3	458	0.069	0.351–0.725	0.725	69.523
Metabolic risk	3	790	0.049	−0.097	0.195	0

BMI, Body Mass Index; I^2^, I-square, measurement for heterogeneity.

Bold values are statistically significant with *p* < 0.05.

### Lifestyle habits

One study (37) examined the effect of a lifestyle intervention on self-reported physical activity (International Physical Activity Questionnaire) and eating habits, but found no significant change in both outcomes. Forsberg et al. (28) observed no change in smoking habits after a lifestyle intervention.

### Sensitivity analysis

In the sensitivity analyses on follow-up duration, intervention type, and sample size, the findings were robust (see Supplementary Information).

### Factors associated with successful implementation

Eight studies (28, 33–37, 39, 40) discussed the implementation of their intervention and/or reflected on barriers and facilitators of successful implementation. In the study of Gyllensten et al. (34) and the study of Cabassa et al. (36) [presented in a separate article (41)] a qualitative analysis was done to study implementation factors. In the other studies implementation factors were evaluated in the discussion of their study. Six studies described the attendance rate of the participants, these rates are depicted in Table 2.

Half of the studies discussed factors associated with staff. Looijmans et al. (35) and Cabassa et al. (36) observed large variations between implementation of the intervention between the different teams and Looijmans et al. (35) argued that the attitude of staff played an important role. Implementation was complicated if there were conflicts in role definitions, insufficient experience with motivating people for obtaining a healthy lifestyle, and conflicting lifestyle behaviors of the mental health care workers themselves. Other barriers were staff turnover (36), lack of time (34, 35) and lack of knowledge (34). Therefore, Gyllensten et al. (34) argued that designated lifestyle nurses/coaches can be useful for teams for successful implementation of a lifestyle intervention because they can support other team members and act as a source of information.

Moreover, social support and supervised group exercise were reported as important elements for success in overcoming the barriers people with SMI experience from being physically active. Group exercise with involvement of staff, family, or peers, and a devoted coach were reported as being helpful in overcoming the barriers of motivational challenges in three studies (33, 36, 39). Participants found involvement of staff and competition with staff encouraging and experienced social support (33, 34, 39).

In addition, Looijmans et al. (35) discussed that psychoeducation on healthy lifestyle should be combined with exercise for a positive impact on physical health outcomes. Gyllensten et al. (34) argued that a successful lifestyle intervention should not be too complex, as technical difficulties in implementing their computer exercise games were perceived as a major barrier that resulted in very low adherence.

Daumit et al. (40) and Verhaeghe et al. (37) recommended to implement lifestyle interventions into the regular psychiatric rehabilitation program. Daumit et al. (40) argued that this could decrease barriers and increase attendance for participants, as they already often attend the psychiatric rehabilitation center.

## Discussion

This systematic review and meta-analysis examined the effect of lifestyle interventions on mental and physical health in people with SMI living in SHF. We found weak evidence for the effectiveness of lifestyle interventions in SHF on mental and physical health. A meta-analysis on mental health outcomes was not possible due to the limited number of studies and non-comparable outcome measures. More studies were performed examining physical outcome measures. Although studies reported significant improvement of weight (*n* = 3), BMI (*n* = 1), 6MWT (*n* = 1) and metabolic criteria (*n* = 2), the meta-analysis only showed a statistically significant reduction in waist circumference, with small effect size (Hedges *g* < 0.20). Reviewing factors involved with the implementation of lifestyle interventions showed that most successfully implemented interventions were multidisciplinary and integrated into standard care.

Our results are in contrast with previous systematic reviews that included patients from various clinical trials and hospital settings. These systematic reviews concluded that lifestyle interventions are effective in improving mental health and quality of life in people with schizophrenia and major depression (13, 15, 18). This discrepancy may be due to the to the different participant population (general SMI population *vs.* people with SMI living in SHF) and low number and relatively low quality of the studies included in the current analysis. Compared to a hospital setting, staffing levels are lower in SHF and implementation in the living environment may be (even) more difficult, as evidenced by the low attendance rates in most of the included studies (5 to 72%). There is often not a structured day program in which healthy lifestyle components can be implemented, as is common in hospital settings. In addition, there is less guidance for individual clients to motivate them to engage in healthy lifestyle activities compared to inpatient settings. Moreover, compared to outpatient settings, patients often face more difficulties in mental health and social functioning and may experience more barriers to participating in lifestyle interventions.

In our meta-analysis, we found that lifestyle interventions only significantly decreased waist circumference and found no difference for other physical outcome measures. Previous meta-analyses have shown conflicting effects of lifestyle interventions on physical health outcomes in people with SMI in the general community (12, 21, 42). Speyer et al. (42) found no significant difference in weight after lifestyle interventions, while Vancampfort et al. (12) found a decrease in weight, waist circumference and BMI (although the latter was only due to individual lifestyle advice and not to exercise interventions).

Studies that discussed factors associated with successful implementation of lifestyle interventions (28, 33–37, 39, 40) mainly mentioned factors related to staff (e.g., own lifestyle, attitude, conflicts in role definition and other tasks), social support, adequate supervision, knowledge of both staff and patients (i.e., psycho-education) and the importance of achievable goal setting and a multidisciplinary, integrated, and multi-component approach as essential factors. This is in line with previous research on implementation barriers and facilitators in inpatients (26) and outpatients [Hassan et al., (27)], and recent recommendations on implementation of lifestyle interventions (18, 21). To successfully and sustainably implement lifestyle interventions for people with SMI living in SHF, we emphasize the importance of a multidisciplinary, highly integrated, and multi-component approach with high support of staff, peers and family, and integration into daily rehabilitation care, so that adherence improves and dropout rates decrease.

### Strengths and limitations

The greatest strength of this study is that it provides an up-to-date and extensive overview of the literature on the outcomes of lifestyle interventions in people with SMI living in SHF and provides a review of factors associated with successful implementation of lifestyle interventions. Previous meta-analyses (12–15, 21, 42) did review the literature on lifestyle interventions for people with SMI, but did not specifically address people living in SHF. However, studies conducted in this setting may provide new insights and be of special interest, as it may present a unique opportunity for the implementation of lifestyle interventions, given the semi-intensive guidance in the direct environment of patients and the close contact with mental health professionals. By thoroughly reviewing the factors associated with successful implementation of different lifestyle interventions, we contribute to the literature on implementation science of lifestyle interventions in clinical practice (43, 44). This is important as evidence on this topic is limited and essential for implementing effective and sustainable lifestyle interventions. Another strength is that the participants in the included studies are diagnosed with a broad range of psychiatric diagnoses and do well represent the “real-world” SMI population.

This study also has some limitations. First, three of the nine included studies also included participants not living in SHF. We requested data from the authors to analyze only the participants living in SHF, but only Looijmans et al. (35) responded. To correct for this potential bias in the analysis, we included only the number of participants living in SHF. Second, the methodological quality of three studies was rated as high concern of bias, mostly because of missing outcome data. The study by Gyllensten et al. (34) was also excluded from the quantitative analyses, because the intervention was hardly carried out due to personnel problems. As a result, the effectiveness of the study could not be reliably estimated. Third, the study of Daumit et al. (40) did not report p-values for systolic and diastolic blood pressure, total cholesterol, HDL cholesterol, triglycerides, and waist circumference. Therefore, we used a p-value of 0.049 for these outcome measures as confidence intervals were small. Fourth, a wide range of lifestyle interventions was included in the analysis which could have led to high heterogeneity in the analysis, we therefore conducted a sensitivity analysis in which results were similar. Fifth, four of the studies had low study samples (*N* < 50), which might have led to underestimation of the results because these studies were underpowered. Finally, it is important to note that the protocol of this study has not been registered prospectively.

### Future research

Previous research on the effect of lifestyle interventions on mental health has shown promising results (18) and future research should be performed in this specific setting of people with SMI living in SHF, as we could now only identify three studies examining this effect. Furthermore, future studies should focus more on the role of SHF staff, as high involvement could provide an opportunity to improve attendance and dropout rates. They can play an important role in overcoming barriers such as lack of motivation due to negative symptoms, anxiety, and lack of social support, as they are an important part of the daily living environment and can motivate patients, provide social support and facilitate a supportive environment for making healthy choices (45). In addition, only two studies examined the effect of lifestyle interventions on cardiorespiratory fitness (36, 39). Physical health outcomes as cardiorespiratory fitness may be more relevant for future lifestyle studies in people with SMI since there are direct dose-response relations between cardiorespiratory fitness and all-cause mortality (46) and associations between improvement of cardiorespiratory fitness and improvements in mental health (47).

In conclusion, we found limited evidence for the effectiveness of lifestyle interventions in SHF on physical health, but could only base this on the outcomes of nine studies. To reliably examine the effects on mental and physical health more studies with high involvement of staff and participants are needed.

## Data availability statement

The original contributions presented in this study are included in the article/Supplementary material, further inquiries can be directed to the corresponding author.

## Author contributions

LK: study design, retrieving, processing and analyzing data, and drafting the manuscript. MH: study design, supporting retrieving, processing and analyzing data, and supporting drafting of the manuscript. JD and WC: study design, supporting analyzing data, and drafting of the manuscript. All authors critically revised the manuscript and approved the final version.
