# Supplementary material for: Lifestyle interventions for people with a severe mental illness living in supported housing: A systematic review and meta-analysis
Source: Front Psychiatry. 2022 Oct 28;13:966029. doi: 10.3389/fpsyt.2022.966029 (PMC9650385; doi:10.3389/fpsyt.2022.966029)

**Supplementary Information**

**Sensitivity analyses.**

**WAIST CIRCUMFERENCE**

**Follow up duration:**


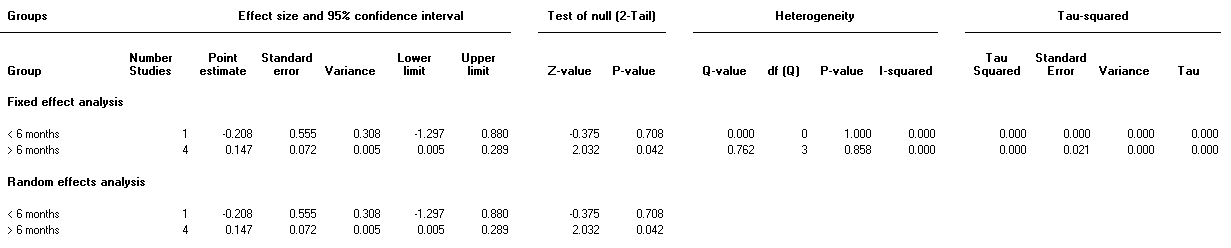


**Exercise in intervention yes/no:**


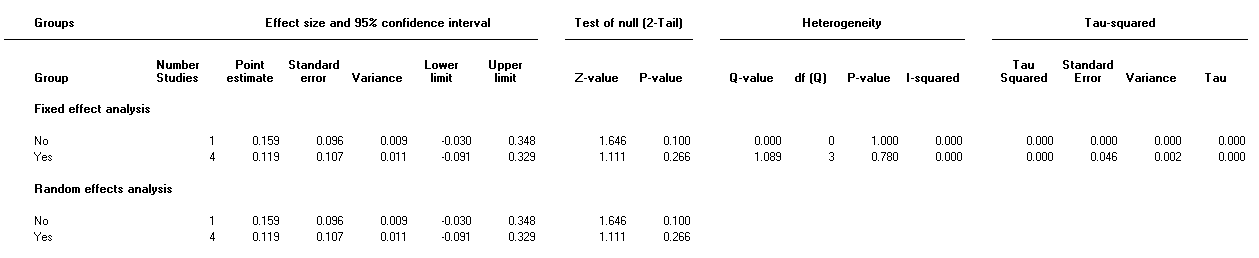


**Sample size >75 = big:**


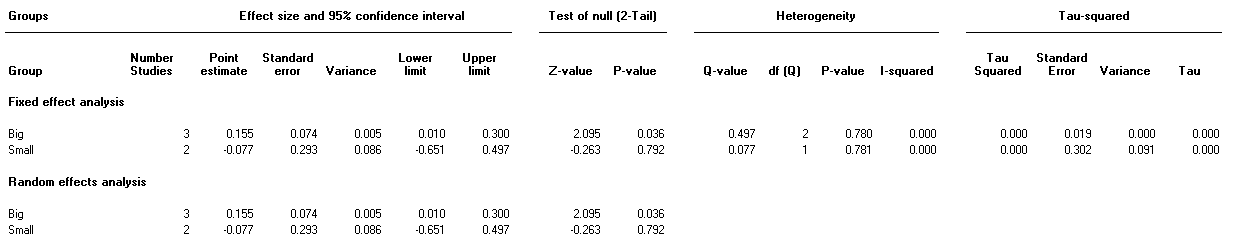


**BMI**

**Follow up duration:**


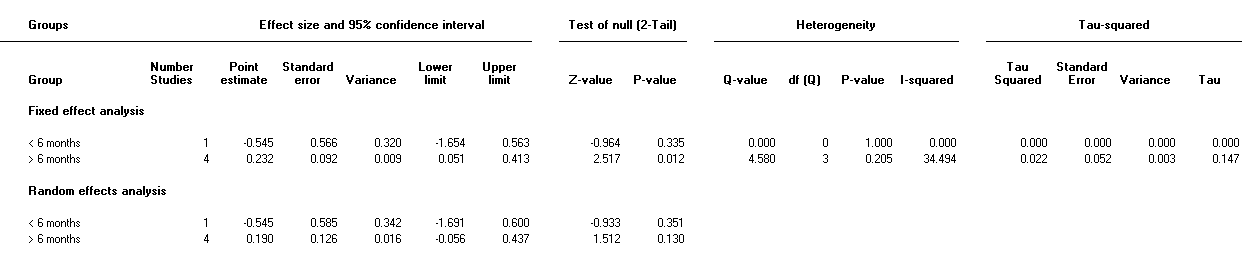


**Exercise in intervention yes/no**


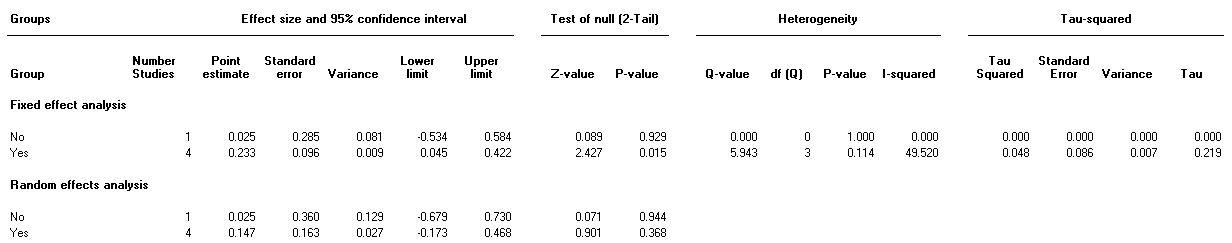


**Sample size >75 = big:**


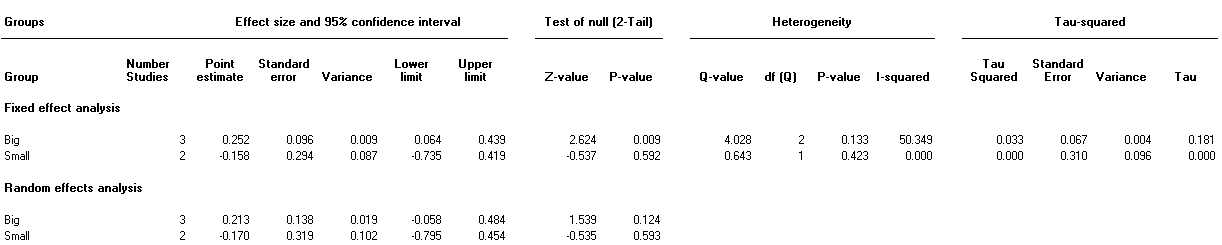


**WEIGHT**

**Follow up duration:**


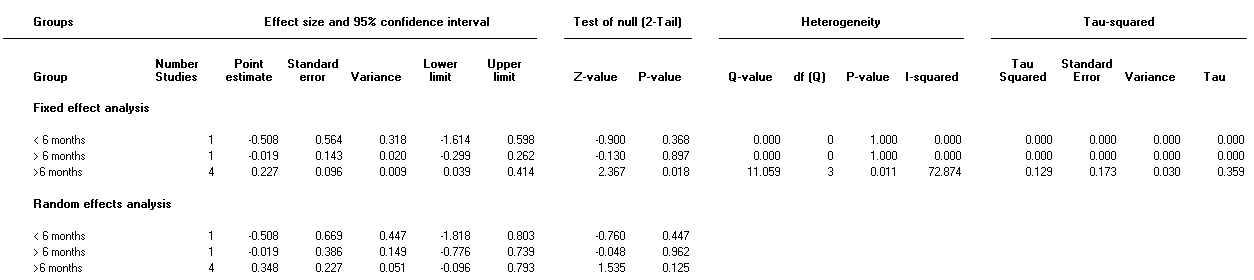


**Exercise in intervention yes/no:**


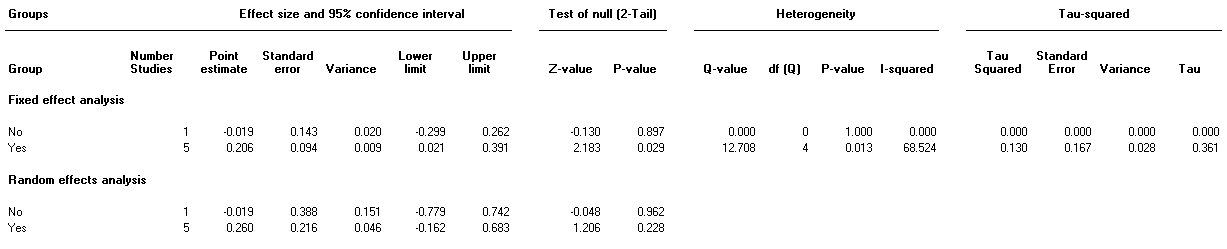


**Sample size, >75 = big:**


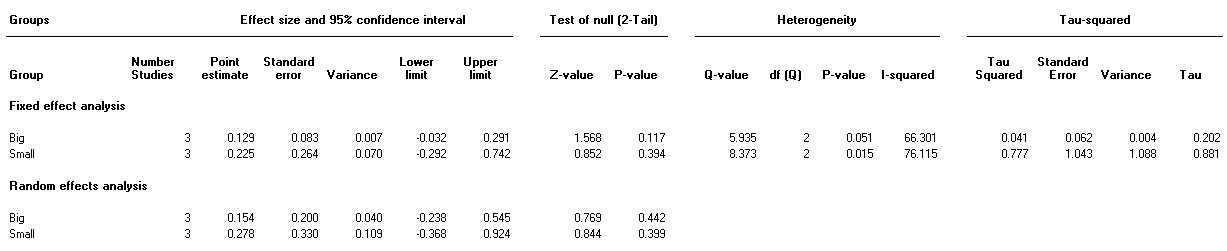


**BLOOD PRESSURE SYSTOLIC**

**Follow up duration:**


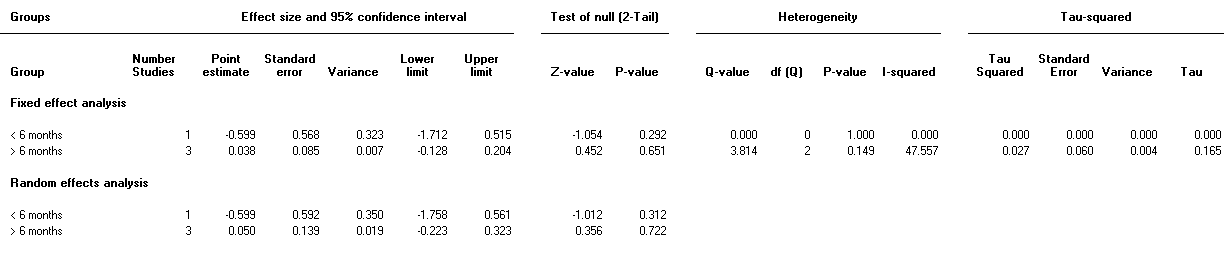


**Exercise in intervention yes/no:**


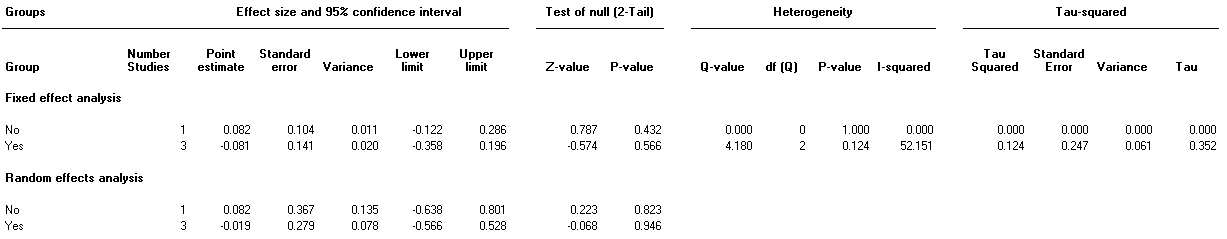


**Sample size. > 75 = big:**


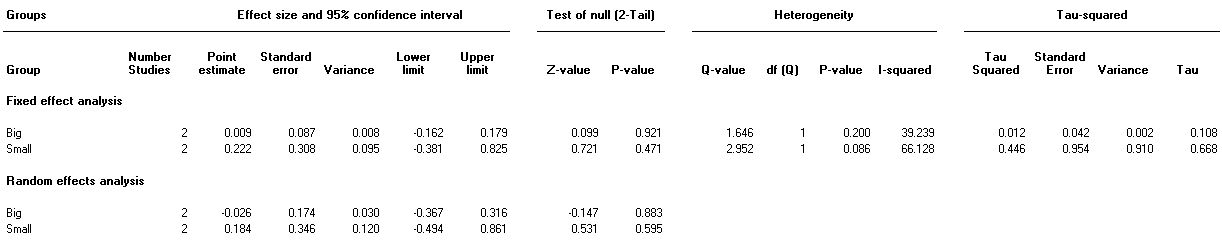


**TRIGLYCERIDES**

**Follow up duration:** all included studies had a long follow up duration, so no sensitivity analysis has been performed.

**Sample size. >75 =big:**


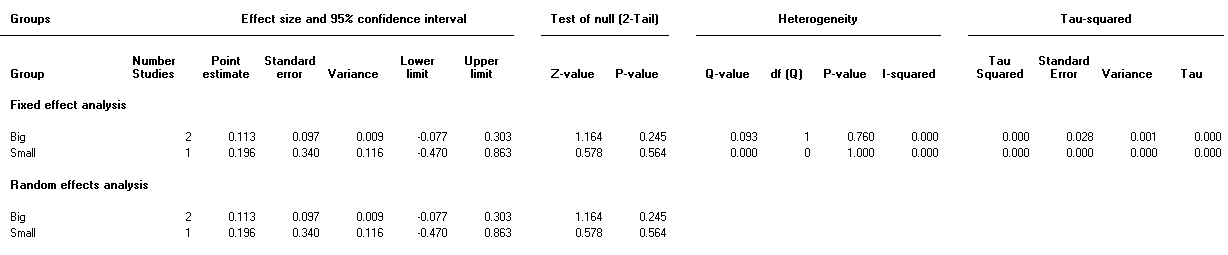


**Exercise in intervention yes/no:**


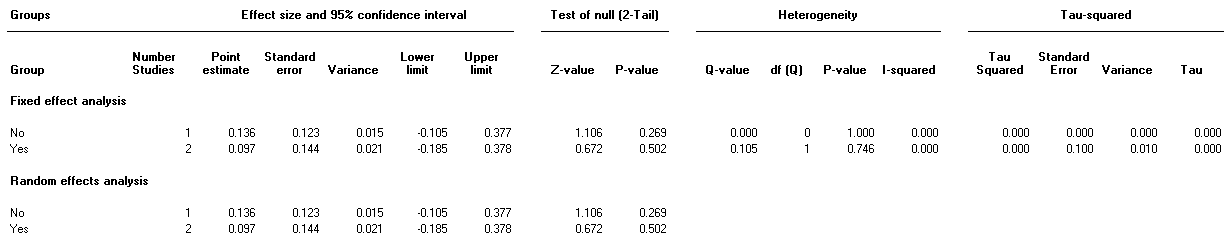


**HDL**

**Follow up duration:** all included studies had a long follow up duration, so no sensitivity analysis has been performed.

**Exercise in intervention yes/no:**


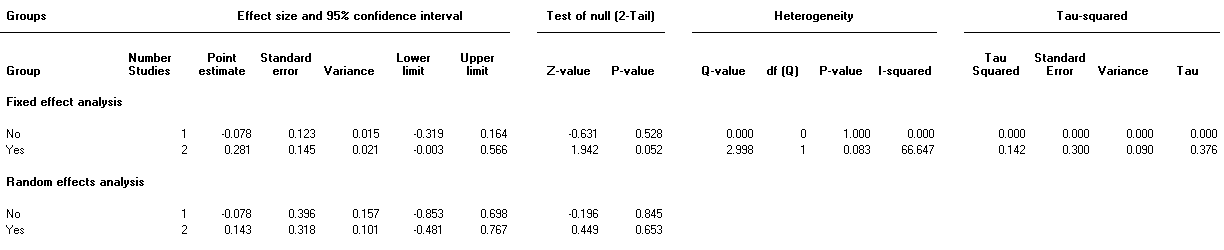


**Sample size. > 75 = big:**


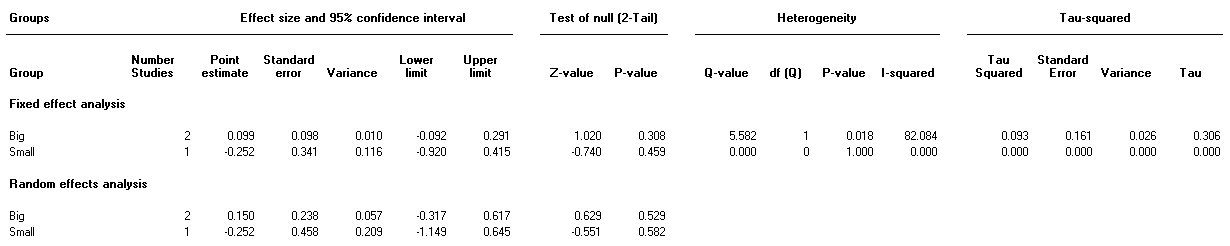


**METABOLIC RISK**

**Follow up duration:** all included studies had a long follow up duration, so no sensitivity analysis has been performed.

**Exercise in intervention yes/no:**


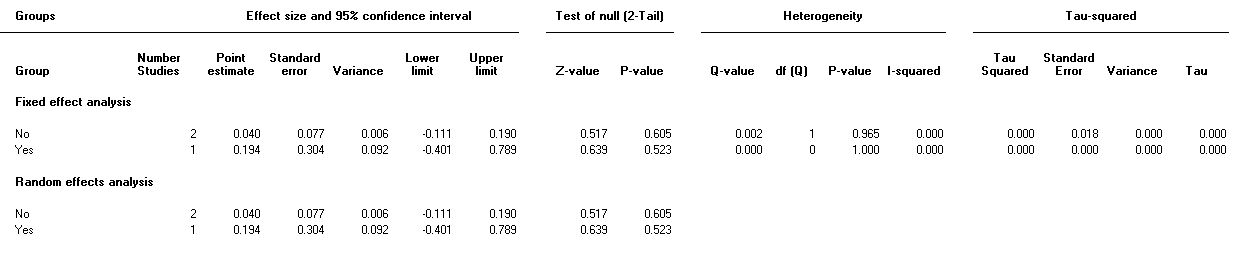


**Sample size. >75 = big:**


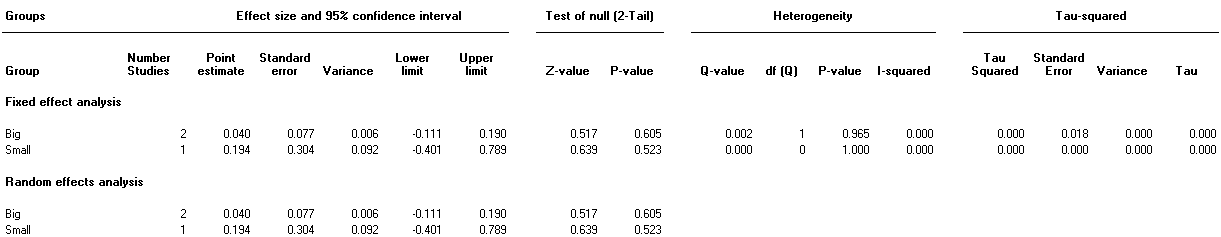

Supplement: Supplementary file 1 [file Data_Sheet_1.docx]
